# A Compendium of Osteology, Being a Systematic Treatise on the Bones of the Human Body; Designed for the Use of Students. To Which Is Subjoined, an Improved Method of Preparing Bones for Osteological Purposes

**Published:** 1835-01-01

**Authors:** 


*A Compendium of Osteology, being a Systematic Treatise on the Bones of the Human Body; designed for the Use of Students. To which is subjoined, an improved Method of Preparing Bones for Osteological Purposes.* By GEORGE WITT, M.D., Physician to the General Infirmary, Bedford.—London and Bedford. 4to. pp. 72.

THIS work will be useful to students. Dr. Witt tells us that his mode of teaching osteology "consists in taking up each bone in succession, and placing and retaining it in one given and fixed position, until the different parts presented to the eye, whether processes, foramina, or grooves, are *read off* in this lucid order and succession. The bone is then turned, as ther instances recorded of the immense sacrifice of property upon a given axis, to bring into sight a fresh collection of parts."

"In practice, the bone, the os occipitis for instance, should be held steadily with the left hand, presenting to view the first-mentioned part in the table; the right hand should hold a probe, or some sharp instrument, to point out the parts. In the occiput the tubercle is the first part to be noticed; hence a posterior aspect of the bone is chosen: in this view we have exposed the first six parts in the table; then, by moving the bone a little, so as to bring the condyles uppermost, the remaining three parts on the external surface will be exposed: the bone must next be turned round to its internal surface, which turn alone will bring into view the remaining seven parts of the os occipitis." (*Preface*, p. iii.)

We have extracted these directions, as we think they may prove useful even to students who do not possess Dr. Witt's book. The following quotation, being a part of our author's description of the os occipitis, will show his method of applying these rules.

| | |
|---|---|
| "OS OCCIPITIS. | *Deriv.* Occiput, ex ob et caput; the hinder part of the head. *Synonyma*, os Basilare, os Proræ, os Memoriæ, os Pixidis, os Nervosum, &c. Like the os frontis this is a single bone, therefore many of the parts occur double. |
| *Situation.* | Posteriorly and inferiorly with respect to the other bones of the cranium. |
| *Connexion.* | With 6 bones; viz. above, with the parietal bones by the lamboidal suture; laterally, with the temporal bones by the additamenta of that suture; in front, with the sphenoid bone at its basilar process; and below, with the first vertebræ of the neck by ginglimus. |
| *Use* ...... | Defends and supports the posterior lobes of the cerebrum, contains the cerebellum, transmits the medulla oblongata, and various vessels and nerves. |
| *External Surface.* | Is irregularly convex, and internal surface irregularly concave. |
| TUBERCLE ........................ | *Tuber Occipitalis.* In the centre of the superior part of the bone, to which the ligamentum nuchæ is attached. In some instances this is a sharp projecting process, in others, there is merely a thickening at this part. |
| PERPENDICULAR SPINE......... | *Spina Occipitalis.* Descending in a straight line from the tubercle, and situated between the muscles of the opposite sides. |
| SUPERIOR TRANSVERSE RIDGE | *Arcus Transversalis Superior.* Extending across the bone, for the attachment of muscles. |
| INFERIOR TRANSVERSE RIDGE | *Arcus Transversalis Inferior.* A little below the former, also for the attachment of muscles. |
| THE OCCIPITAL FORAMEN...... | *Foramen Magnum.* For the transmission of the medulla oblongata, the exit e cranio of the anterior arteries of the medulla spinalis, and the entrance into the skull of the nervi accessorii, and vertebral arteries. |

| | |
|---|---|
| 2 Condyles .................... | *Processus Condyloidei.* Situated on each side of the foramen magnum towards its front part, by which this bone is connected with the atlas, around the condyles there are ridges for the attachment of the capsules of the joints, and various depressions for the attachment of strong ligaments, for the security of the articulation." (P. 8.) |

We hope that a second edition will give our author an opportunity of correcting some of his Greek and Latin words; thus, at p. 39, he has "the pubis" for the pubes or os pubis, and "symphisis" for symphysis; and at p. 40 we have so monstrous and impossible a word as χοχχυξ:—impossible, we say, for the delicate ear of the Greeks, so far from allowing two χs to come together, would not permit them to commence two contiguous syllables. If physicians wish to be considered members of a learned profession, these things are not unworthy of their attention; if they are satisfied to be mere traders in prescriptions, the case, we confess, is different.

Our author's *Observations on the Mode of Preparing Bones for Osteological Purposes* have an air of truth and genuineness, and we therefore extract them entire.

"Having been repeatedly solicited by various friends, who have seen the bones in my collection, to explain to them the manner in which they were prepared, I now gladly avail myself of an opportunity of making it more generally known.

"About twelve years since my attention was particularly directed to this subject, by finding that some bones which I had macerated were unusually white, and free from smell. I continued for several subsequent years to macerate and prepare bones, as I conceived, precisely upon the same plan as that in which the maceration had been so successful; but, although some proved tolerably white, the majority were cleaned with much difficulty, and when the ligamentous attachments were removed by dint of hard scraping, the bones were ever after yellow at the extremities, and had a more or less offensive smell. After much thought on the subject, I could not discover wherein my method differed from that in general use, but the preparation of another skeleton, about four years back, furnished me with materials whereupon to build something like a tangible theory; and this theory having been verified by repeated subsequent trials, I feel confident in recommending the practice founded upon it, although perhaps the reasons advanced may not be altogether conclusive. Two words, however, if properly understood, will furnish all the information that is necessary, viz. *Uninterrupted Putrefaction*; for, if the putrefactive process be in any way interrupted, the bones will never be clean. In order to obtain this end, the following directions must be scrupulously observed: It is desirable to get the animal, of which a skeleton is to be made, as few hours after death as possible, while the blood is in an almost fluid state, and having taken off the muscles tolerably clean, and separated every bone, they should be immediately thrown into cold water; the water should be changed every twelve hours for three or four days, until it becomes no longer tinged with blood. A tub, or large earthenware vessel, must then be procured; if a tub, it must be well made, to secure it from leaking during the long period required for maceration, and of such a size as to hold a sufficient quantity of water over and above that which covers the bones, to allow for the waste by evaporation. Evaporation entails two difficulties, for if it go so far as to leave the ends of some of the longer bones projecting out of the water, they immediately become quite black, and if fresh water be added to cover them, the whole putrefactive process is arrested: hence the vessel must stand in some covered outhouse, secure from the admission of rain, and from the danger of the water being drunk by rats. So far as I have observed, the vessels should be merely lightly covered over, as a certain access of air appears necessary; for, on one occasion, being without a convenient place, I buried some tubs during the usual period, when the bones proved the most offensive that I ever prepared, and it was an endless task to get off the ligaments.

"Should all go on successfully, and the process be in no way interrupted, either by evaporation or by leakage, in the space of about six months the bones may be washed perfectly clean with a common brush, the ligaments and muscular attachments may be pushed off like a cake from the ends of the bones, and then, if they be held up, a thick fluid will be seen to exude through every aperture from the interior, proving that the putrefactive process has gone on in the interior with the same happy result as on the exterior. It is hardly necessary to observe, that the internal and external putrefaction must go hand in hand in order to procure a clean bone, and this I apprehend to be the general source of difficulty. A vertical section of any of the cylindrical bones in my collection exhibits the interior even whiter than the exterior; and the cancellous structure is a beautiful white net-work, unsoiled by any medullary matter. After the bones have been well brushed, they should be soaked in clean water twenty-four hours, and then carefully cleaned with a scalpel from all ligamentous and cartilaginous matter that may be found still adhering, but the bone should in no way be scraped more than is absolutely necessary, as it is deprived of all its minute processes and distinctive characteristics. The long cylindrical bones should be placed upright upon their extremities for a short time, in order to allow of the entire escape of the medullary fluid. To cleanse them from any oleaginous matter, whether external or internal, I have generally soaked them for the next forty-eight hours in a solution of subcarbonate of potass, in the proportion of about a pound to each gallon of water, after which they should again be well washed and left for a short time in a large quantity of clean water, and then wiped with a dry cloth. The bones after this should be carefully laid upon a clean deal board, and exposed for a few days and nights in the open air, taking the precaution to turn them now and then.

"As to the exact period for maceration, six months has been stated as a general time; but this is found to vary, as a set of bones will macerate in almost half the time during the summer months to what will be required in the winter. The bones of small animals, and of birds also, require a comparatively short period; and it may be observed, that the bones of the ruminating class of animals always macerate more speedily than those of the carnivorous." (P. 69.)

